# Analysis of Multilocus Sequence Typing and Virulence Characterization of *Listeria monocytogenes* Isolates from Chinese Retail Ready-to-Eat Food

**DOI:** 10.3389/fmicb.2016.00168

**Published:** 2016-02-16

**Authors:** Shi Wu, Qingping Wu, Jumei Zhang, Moutong Chen, Weipeng Guo

**Affiliations:** ^1^Guangdong Institute of Microbiology, State Key Laboratory of Applied Microbiology Southern China, Guangdong Provincial Key Laboratory of Microbial Culture Collection and Application, Guangdong Open Laboratory of Applied MicrobiologyGuangzhou, China; ^2^School of Bioscience and Bioengineering, South China University of TechnologyGuangzhou, China

**Keywords:** *Listeria monocytogenes*, MLST, *inlA*, PMSC, virulence genes, epidemic clones

## Abstract

Eighty *Listeria monocytogenes* isolates were obtained from Chinese retail ready-to-eat (RTE) food and were previously characterized with serotyping and antibiotic susceptibility tests. The aim of this study was to characterize the subtype and virulence potential of these *L. monocytogenes* isolates by multilocus sequence typing (MLST), virulence-associate genes, epidemic clones (ECs), and sequence analysis of the important virulence factor: internalin A (inlA). The result of MLST revealed that these *L. monocytogenes* isolates belonged to 14 different sequence types (STs). With the exception of four new STs (ST804, ST805, ST806, and ST807), all other STs observed in this study have been associated with human listeriosis and outbreaks to varying extents. Six virulence-associate genes (*inlA, inlB, inlC, inlJ, hly*, and *llsX*) were selected and their presence was investigated using PCR. All strains carried *inlA, inlB, inlC, inlJ*, and *hly*, whereas 38.8% (31/80) of strains harbored the listeriolysin S genes (*llsX*). A multiplex PCR assay was used to evaluate the presence of markers specific to epidemic clones of *L. monocytogenes* and identified 26.3% (21/80) of ECI in the 4b-4d-4e strains. Further study of *inlA* sequencing revealed that most strains contained the full-length InlA required for host cell invasion, whereas three mutations lead to premature stop codons (PMSC) within a novel PMSCs at position 326 (GAA → TAA). MLST and *inlA* sequence analysis results were concordant, and different virulence potentials within isolates were observed. These findings suggest that *L. monocytogenes* isolates from RTE food in China could be virulent and be capable of causing human illness. Furthermore, the STs and virulence profiles of *L. monocytogenes* isolates have significant implications for epidemiological and public health studies of this pathogen.

## Introduction

*Listeria monocytogenes* is a gram-positive, facultative intracellular bacterium that is responsible for listeriosis. Listeriosis can cause meningitis, newborn septicemia, encephalomyelitis, or even death in humans, especially in the elderly, pregnant women, or newborn. Every year, ~1591 cases of listeriosis in humans are report, with a 19% case-fatality rate in the United States (Scallan et al., 2011). In the European Union, a total of 1763 confirmed human cases of listeriosis (notification rate of 0.44 cases per 100,000 population) were reported in 2013 (EFSA, 2015). As an important foodborne pathogen, it is widespread in nature and lives naturally in plants and soil environments. Its ability to survive and grow over a wide range of environmental conditions, including refrigeration temperatures, high salt concentration and low pH, makes it a potential hazard in foods (Ryser and Marth, 2007).

Differences in virulence between *L. monocytogenes* strains may also influence infection and clinical outcome. Some strains are highly pathogenic and sometimes deadly, whereas others are less virulent or even avirulent and produce little harm in the host (Olier et al., 2002). Several methods have been used to differentiate *L. monocytogenes* strains. Based on somatic (O) and flagellar (H) antigens represents a conventional approach to understanding *L. monocytogenes* isolates ecological distribution and epidemiology. However, there are 13 serotypes and only four serotype (1/2a, 1/2b, 1/2c, and 4b) cause almost all cases of listeriosis in human. To further discriminate these strains, numerous molecular methods have been developed for epidemiological investigations and of help for the surveillance and control of listeriosis. Multilocus sequence typing (MLST) method is one of the most robust tools for investigating the global epidemiology of microbial populations (Sullivan et al., 2005). Based on the sequencing of seven housekeeping genes, it is highly discriminatory and provides unambiguous results that can be comparable directly among laboratories via the internet. In recent years, MLST has evolved to the reference method for global epidemiology and population biology (Maiden, 2006; Ragon et al., 2008).

Certain virulence and virulence-associated genes also play very important roles in intracellular survival, cell-to-cell spread, and virulence of *L. monocytogenes*. The presence of a number of virulence factors such as surface-associated internalins, listeriolysin O, and listeriolysin S (LLS) in *L. monocytogenes* significantly regulates its pathogenicity (Cotter et al., 2008; Shen et al., 2013). Of which, internalin A (InlA), encode by *inlA*, is responsible for facilitating the entry of *L. monocytogenes* into nonprofessional phagocytic cells expressing the human isoform of E-cadherin (Lecuit et al., 2001). It has been shown that mutations in *inlA* leading to a premature stop codon (PMSC) significantly reduce the invasion of the strain to human epithelial cells (Nightingale et al., 2005a). In addition, a small number of closely related *L. monocytogenes* clones have caused multiple outbreaks worldwide. These epidemic clones (EC) are divided into seven groups: ECI, ECII and ECIV within serotype 4b, ECIII, ECV and ECVII in serotype 1/2a, and ECVI within serotype 1/2b (Kathariou, 2003; Knabel et al., 2012; Lomonaco et al., 2013).

Since the main food linked to listeriosis outbreaks was ready-to-eat (RTE) food, the consumer had limit opportunities to destroy the pathogen before the food consumed. Therefore, the risk associated with strains isolated from RTE food may be more severe. In our previous study, 80 RTE *L. monocytogenes* isolates were examined (Wu et al., 2015a). In order to determine the potential pathogenic profile and relative risk of these RTE *L. monocytogenes* isolates, this study provide a phylogenetic framework based on MLST analysis of *L. monocytogenes* isolates and evaluate their virulence-genes and EC-specific markers. In addition, we analyzed the full-length sequences of important virulence factor (inlA) to investigate correlations among the MLST types, amino acid sequence of inlA, and virulence potential.

## Materials and methods

### Bacterial isolates

A total of 80 *L. monocytogenes* isolates were collected from retail ready-to-eat food samples in 24 Chinese cities were analyzed, comprising 27 isolates from cold vegetable dishes in sauce, 7 isolates from cold noodles dishes in sauce, 17 isolates from roast chicken, 12 isolates from roast duck, 11 isolates from roast pork, and 5 isolates from pasteurized milk (Table 1). The isolates were obtained between September 2012 and January 2014 according to the GB 4789.30-2010 of food microbiological examination of *L. monocytogenes* (National Food Safety Standards of China) with slight modifications and the most probable number (MPN) method (Gombas et al., 2003). They were identified by Gram stain, catalase, and oxidase tests and Micro ID *Listeria* identification system (Microgen, Camberley, UK). Serovars and antibiotic susceptibility were confirmed in previous study (Wu et al., 2015a). Additionally, 35 isolates were selected for the sequencing analysis of *inlA*, based on MLST profile, molecular serogroup, food origin, and antimicrobial resistance. Each isolate was incubated at 37°C overnight on TSB-YE (Tryptic soy agar with yeast extract). Genomic DNA was extracted using a Genomic DNA Extraction kit (Dongsheng Biotech, Guangzhou, China) according to the manufacturer's instructions. The concentration of genomic DNA was determined at 260 nm using a NanoDrop-ND-1000 UV-Vis Spectrophotometer (Thermo Fisher Scientific, MA, USA).

**Table 1:** **Characteristics of 80 *L. monocytogenes* strains isolated from retail ready-to-eat food in this study**.

**Strain/isolate**	**Strain orgin**	**Sample city**	**Year**	**Serovar^a^**	**Antibiotic susceptibility^a^**	**ST**	***hly***	***inlB***	***inlA***	***inlC***	***inlJ***	***llsX***	**EC**
859-1LM	Cold vegetable dish in sauce	Hefei	2012	1/2a-3a	AMP-CIP-DA	ST-8	+	+	+	+	+	−	−
859-2LM	Cold vegetable dish in sauce	Hefei	2012	1/2b-3b-7	CIP-DA-E-K-RD-S-TE	ST-87	+	+	+	+	+	−	−
859-3LM	Cold vegetable dish in sauce	Hefei	2012	1/2a-3a	DA-RD	ST-8	+	+	+	+	+	−	−
859-4LM	Cold vegetable dish in sauce	Hefei	2012	1/2a-3a	DA	ST-8	+	+	+	+	+	−	−
860-1LM	Cold noodles dishes in sauce	Hefei	2012	1/2b-3b-7	AMP-CIP-DA	ST-87	+	+	+	+	+	−	−
860-2LM	Cold noodles dishes in sauce	Hefei	2012	1/2a-3a	AMP-CIP-DA	ST-8	+	+	+	+	+	−	−
860-3LM	Cold noodles dishes in sauce	Hefei	2012	1/2b-3b-7	CIP-DA	ST-5	+	+	+	+	+	−	−
860-4LM	Cold noodles dishes in sauce	Hefei	2012	1/2a-3a	DA	ST-8	+	+	+	+	+	−	−
959-1LM	Cold vegetable dish in sauce	Wuhan	2012	1/2b-3b-7	DA	ST-87	+	+	+	+	+	−	−
959-2LM	Cold vegetable dish in sauce	Wuhan	2012	1/2a-3a	AMP-KF-C-DA-E-CN-K-RD-S-TE-VA	ST-8	+	+	+	+	+	−	−
959-3LM	Cold vegetable dish in sauce	Wuhan	2012	1/2a-3a	DA	ST-8	+	+	+	+	+	−	−
959-4LM	Cold vegetable dish in sauce	Wuhan	2012	1/2a-3a	DA	ST-8	+	+	+	+	+	−	−
1009-1LM	Cold vegetable dish in sauce	Chengdu	2012	1/2b-3b-7	DA	ST-224	+	+	+	+	+	+	−
1009-2LM	Cold vegetable dish in sauce	Chengdu	2012	1/2b-3b-7	DA	ST-224	+	+	+	+	+	+	−
1009-3LM	Cold vegetable dish in sauce	Chengdu	2012	1/2b-3b-7	E	ST-224	+	+	+	+	+	+	−
1009-4LM	Cold vegetable dish in sauce	Chengdu	2012	1/2b-3b-7	DA	ST-224	+	+	+	+	+	+	−
1059-1LM	Cold vegetable dish in sauce	Kunming	2012	1/2b-3b-7	DA	ST-87	+	+	+	+	+	−	−
1059-2LM	Cold vegetable dish in sauce	Kunming	2012	1/2b-3b-7	SUS	ST-87	+	+	+	+	+	−	−
1059-3LM	Cold vegetable dish in sauce	Kunming	2012	1/2c-3c	SUS	ST-87	+	+	+	+	+	−	−
1059-4LM	Cold vegetable dish in sauce	Kunming	2012	1/2c-3c	SUS	ST-87	+	+	+	+	+	−	−
1111-1LM	Roast chicken	Lanzhou	2012	1/2a-3a	DA	ST-7	+	+	+	+	+	−	−
1111-2LM	Roast chicken	Lanzhou	2012	1/2a-3a	SUS	ST-7	+	+	+	+	+	−	−
1111-3LM	Roast chicken	Lanzhou	2012	1/2a-3a	SUS	ST-7	+	+	+	+	+	−	−
1111-4LM	Roast chicken	Lanzhou	2012	1/2a-3a	SUS	ST-7	+	+	+	+	+	−	−
1159-1LM	Cold vegetable dish in sauce	Haerbin	2012	1/2a-3a	DA	ST-8	+	+	+	+	+	−	−
1159-2LM	Cold vegetable dish in sauce	Haerbin	2012	1/2a-3a	DA	ST-8	+	+	+	+	+	−	−
1159-3LM	Cold vegetable dish in sauce	Haerbin	2012	1/2c-3c	DA	ST-9	+	+	+	+	+	−	−
1159-4LM	Cold vegetable dish in sauce	Haerbin	2012	1/2c-3c	SUS	ST-9	+	+	+	+	+	−	−
1194-1LM	Roast chicken	Haerbin	2012	1/2a-3a	DA	ST-8	+	+	+	+	+	−	−
1194-2LM	Roast chicken	Haerbin	2012	1/2a-3a	DA	ST-8	+	+	+	+	+	−	−
1194-3LM	Roast chicken	Haerbin	2012	1/2b-3b-7	DA-TE	ST-804	+	+	+	+	+	+	−
1194-4LM	Roast chicken	Haerbin	2012	1/2b-3b-7	AMP-KF-C-DA-E-K-RD-S-TE-VA	ST-805	+	+	+	+	+	+	−
1244-1LM	Cold vegetable dish in sauce	Xi'an	2012	1/2b-3b-7	SUS	ST-3	+	+	+	+	+	+	−
1244-2LM	Cold vegetable dish in sauce	Xi'an	2012	1/2b-3b-7	SUS	ST-3	+	+	+	+	+	+	−
1244-3LM	Cold vegetable dish in sauce	Xi'an	2012	1/2b-3b-7	SUS	ST-804	+	+	+	+	+	+	−
1244-4LM	Cold vegetable dish in sauce	Xi'an	2012	1/2b-3b-7	DA	ST-3	+	+	+	+	+	+	−
1309-1LM	Cold vegetable dish in sauce	Beijing	2013	1/2a-3a	SUS	ST-121	+	+	+	+	+	−	−
1309-2LM	Cold vegetable dish in sauce	Beijing	2013	1/2a-3a	SUS	ST-121	+	+	+	+	+	−	−
1329-1LM	Roast pork	Beijing	2013	1/2a-3a	DA-TE	ST-155	+	+	+	+	+	−	−
1329-2LM	Roast pork	Beijing	2013	1/2a-3a	AMP-KF-C-DA-E-CN-K-RD-S-TE-VA	ST-155	+	+	+	+	+	−	−
1330-1LM	Cold vegetable dish in sauce	Beijing	2013	1/2a-3a	DA-TE	ST-155	+	+	+	+	+	−	−
1330-2LM	Cold vegetable dish in sauce	Beijing	2013	1/2a-3a	AMP-KF-C-DA-E-CN-K-RD-S-TE-VA	ST-806	+	+	+	+	+	−	−
1342-1LM	Pasteurized milk	Beijing	2013	1/2a-3a	AMP-KF-C-DA-E-CN-K-RD-S-TE-VA	ST-806	+	+	+	+	+	−	−
1342-2LM	Pasteurized milk	Beijing	2013	1/2a-3a	AMP-KF-C-DA-E-CN-K-RD-S-TE-VA	ST-155	+	+	+	+	+	−	−
1584-1LM	Roast duck	Guangzhou	2013	4b-4d-4e	DA	ST-807	+	+	+	+	+	+	ECI
1584-2LM	Roast duck	Guangzhou	2013	4b-4d-4e	CIP	ST-807	+	+	+	+	+	+	ECI
1584-3LM	Roast duck	Guangzhou	2013	4b-4d-4e	C-CIP	ST-807	+	+	+	+	+	+	ECI
1586-1LM	Roast pork	Guangzhou	2013	4b-4d-4e	SUS	ST-1	+	+	+	+	+	+	ECI
1586-2LM	Roast pork	Guangzhou	2013	4b-4d-4e	SUS	ST-1	+	+	+	+	+	+	ECI
1586-3LM	Roast pork	Guangzhou	2013	4b-4d-4e	DA	ST-1	+	+	+	+	+	+	ECI
1588-1LM	Roast chicken	Guangzhou	2013	4b-4d-4e	DA	ST-1	+	+	+	+	+	+	ECI
1588-2LM	Roast chicken	Guangzhou	2013	4b-4d-4e	CIP	ST-807	+	+	+	+	+	+	ECI
1588-3LM	Roast chicken	Guangzhou	2013	4b-4d-4e	CIP	ST-807	+	+	+	+	+	+	ECI
1634-1LM	Roast duck	Guangzhou	2013	4b-4d-4e	SUS	ST-1	+	+	+	+	+	+	ECI
1634-2LM	Roast duck	Guangzhou	2013	4b-4d-4e	CIP	ST-1	+	+	+	+	+	+	ECI
1634-3LM	Roast duck	Guangzhou	2013	4b-4d-4e	CIP	ST-1	+	+	+	+	+	+	ECI
1760-1LM	Cold noodles dishes in sauce	Zhanjiang	2013	1/2a-3a	CIP	ST-8	+	+	+	+	+	−	−
1760-2LM	Cold noodles dishes in sauce	Zhanjiang	2013	1/2a-3a	CIP-DA	ST-8	+	+	+	+	+	−	−
1760-3LM	Cold noodles dishes in sauce	Zhanjiang	2013	1/2a-3a	SUS	ST-8	+	+	+	+	+	−	−
1834-1LM	Roast duck	Shaoguan	2013	4b-4d-4e	CIP	ST-1	+	+	+	+	+	+	ECI
1834-2LM	Roast duck	Shaoguan	2013	4b-4d-4e	CIP	ST-1	+	+	+	+	+	+	ECI
1834-3LM	Roast duck	Shaoguan	2013	4b-4d-4e	CIP	ST-1	+	+	+	+	+	+	ECI
1836-1LM	Roast chicken	Shaoguan	2013	4b-4d-4e	SUS	ST-1	+	+	+	+	+	+	ECI
1836-2LM	Roast chicken	Shaoguan	2013	4b-4d-4e	SUS	ST-1	+	+	+	+	+	+	ECI
1836-3LM	Roast chicken	Shaoguan	2013	4b-4d-4e	SUS	ST-1	+	+	+	+	+	+	ECI
1838-1LM	Roast pork	Shaoguan	2013	4b-4d-4e	SUS	ST-1	+	+	+	+	+	+	ECI
1838-2LM	Roast pork	Shaoguan	2013	4b-4d-4e	CIP	ST-1	+	+	+	+	+	+	ECI
1838-3LM	Roast pork	Shaoguan	2013	4b-4d-4e	CIP	ST-1	+	+	+	+	+	+	ECI
2311-1LM	Roast pork	Xiamen	2013	1/2c-3c	SUS	ST-9	+	+	+	+	+	−	−
2311-2LM	Roast pork	Xiamen	2013	1/2c-3c	SUS	ST-9	+	+	+	+	+	−	−
2311-3LM	Roast pork	Xiamen	2013	1/2c-3c	SUS	ST-9	+	+	+	+	+	−	−
2339-1LM	Roast duck	Xiamen	2013	1/2a-3a	DA	ST-8	+	+	+	+	+	−	−
2339-2LM	Roast duck	Xiamen	2013	1/2a-3a	DA	ST-8	+	+	+	+	+	−	−
2339-3LM	Roast duck	Xiamen	2013	1/2a-3a	DA	ST-8	+	+	+	+	+	−	−
2408-1LM	Pasteurized milk	Haikou	2014	1/2a-3a	DA	ST-8	+	+	+	+	+	−	−
2408-2LM	Pasteurized milk	Haikou	2014	1/2a-3a	SUS	ST-8	+	+	+	+	+	−	−
2408-3LM	Pasteurized milk	Haikou	2014	1/2a-3a	C	ST-8	+	+	+	+	+	−	−
2436-1LM	Roast chicken	Haikou	2014	1/2a-3a	DA	ST-8	+	+	+	+	+	−	−
2436-2LM	Roast chicken	Haikou	2014	1/2a-3a	SUS	ST-8	+	+	+	+	+	−	−
2436-3LM	Roast chicken	Haikou	2014	1/2a-3a	SUS	ST-8	+	+	+	+	+	−	−

a *SUS, susceptibility; P, penicillin G (5U); AMP, ampicillin (10 μg); KF, Cephalothin (30 μg); C, chloramphenicol (30 μg); CIP, Ciprofloxacin (5 μg); DA, Clindamycin (2 μg); E, Erythromycin (15 μg); CN, gentamicin (10 μg); K, Kanamycin (30 μg); MEZ, Mezlocillin (30 μg); RD, Rifampicin (5 μg); S, Streptomycin (25 μg); TE, tetracycline (30 μg); VA, Vancomycin (30 μg). The serovars and antibiotic susceptibility of L. monocytogenes isolates were detected in previous study by Wu et al. (2015a)*.

### Multilocus sequence typing

The MLST scheme used to characterize *L. monocytogenes* isolates is based on the sequence analysis of the following seven housekeeping genes: *abcZ* (ABC transporter), *bglA* (beta-glucosidase), *cat* (catalase), *dapE* (succinyl diaminopimelate desuccinylase), *dat* (D-amino acid aminotransferase), *ldh* (L-lactate dehydrogenase), and *lhkA* (histidine kinase) (Salcedo et al., 2003). The PCR amplification conditions were as follow: an initial cycle of 94°C for 4 min; 35 cycles of 94°C for 30 s, 52°C for 30 s (45°C for *bglA*), 72°C for 2 min, and a final extension at 72°C for 10 min. The DNA fragments were purified using a PCR purification kit (Qiagen, Genman) and were sequenced in each direction with Big Dye fluorescent terminators on an ABI3730XL sequencer (Applied BioSystems).

### Determination of virulence genes and epidemic clone

The isolates were identified using duplex PCR detection containing *hly* (707 bp) and *inlB* (367 bp) genes, which are specific to *L. monocytogenes*, as previously described (Xu et al., 2009). Multiplex PCR (Liu et al., 2007) was used to determine the presence of the virulence genes *inlA, inlC*, and *inlJ*. The *llsX* gene was detected by PCR assays to identify the LLS-positive *L. monocytogenes* strains (Clayton et al., 2011). Presumptive major ECs (ECI, ECII, and ECIII) were found in the isolates as described previously (Chen and Knabel, 2007). All primers and PCR conditions are presented in Supplementary Table 1.

### *inlA* gene sequencing

The 2400 bp long *inlA* gene was sequenced in 35 isolates representing the clonal diversity of *L. monocytogenes*. External primers were used to amplification covering the whole *inlA* ORF and internal primers for sequencing (Supplementary Table 2). The *inlA* sequences were assembled using Seqman (DNASTAR, Lasergene). Mutation types were determined according to the site of mutation that leads to PMSC in *inlA* (Nightingale et al., 2005a) and by comparing the obtained *inlA* sequence data to that of the *L. monocytogenes* EGDe reference strain (Glaser et al., 2001).

### Data analysis

For each MLST locus, an allele number was given to each distinct sequence variant, and a distinct sequence type (ST) number was attributed to each distinct combination of alleles among the seven genes. Sequence types (STs) were determined by using the *L. monocytogenes* MLST database (http://bigsdb.web.pasteur.fr/listeria/listeria.html). Sequence Type Analysis and Recombinational Tests software (S.T.A.R.T. ver.2; http://pubmlst.org/software/analysis/start2) was used to analyze the data of MLST.

Nucleotide diversity (π, average pairwise nucleotide difference/site; and *k*, average pairwise nucleotide difference/sequence), number of polymorphic sites, number of mutations, number of alleles, G+C content, Tajima's *D* test for neutrality, number of synonymous mutations (*Ks*), number of nonsynonymous mutations (*Ka*), and the *Ks*/*Ka* ratios [the number of nonsynonymous substitutions/nonsynonymous site (*Ks*) to the number of synonymous substitutions/synonymous site (*Ka*)] with a Jukes and Cantor correction were calculated using DnaSP version 5.10.01. Sequence analysis of *inlA* was performed using the ClustalX algorithm (version 1.83) (Thompson et al., 1997), which was followed by phylogenetic analysis using the maximum likelihood algorithm in MEGA 6 (version 6.05) (Tamura et al., 2011).

## Results

### Multilocus sequence typing of *L. monocytogenes* isolates

**Figure 1:**
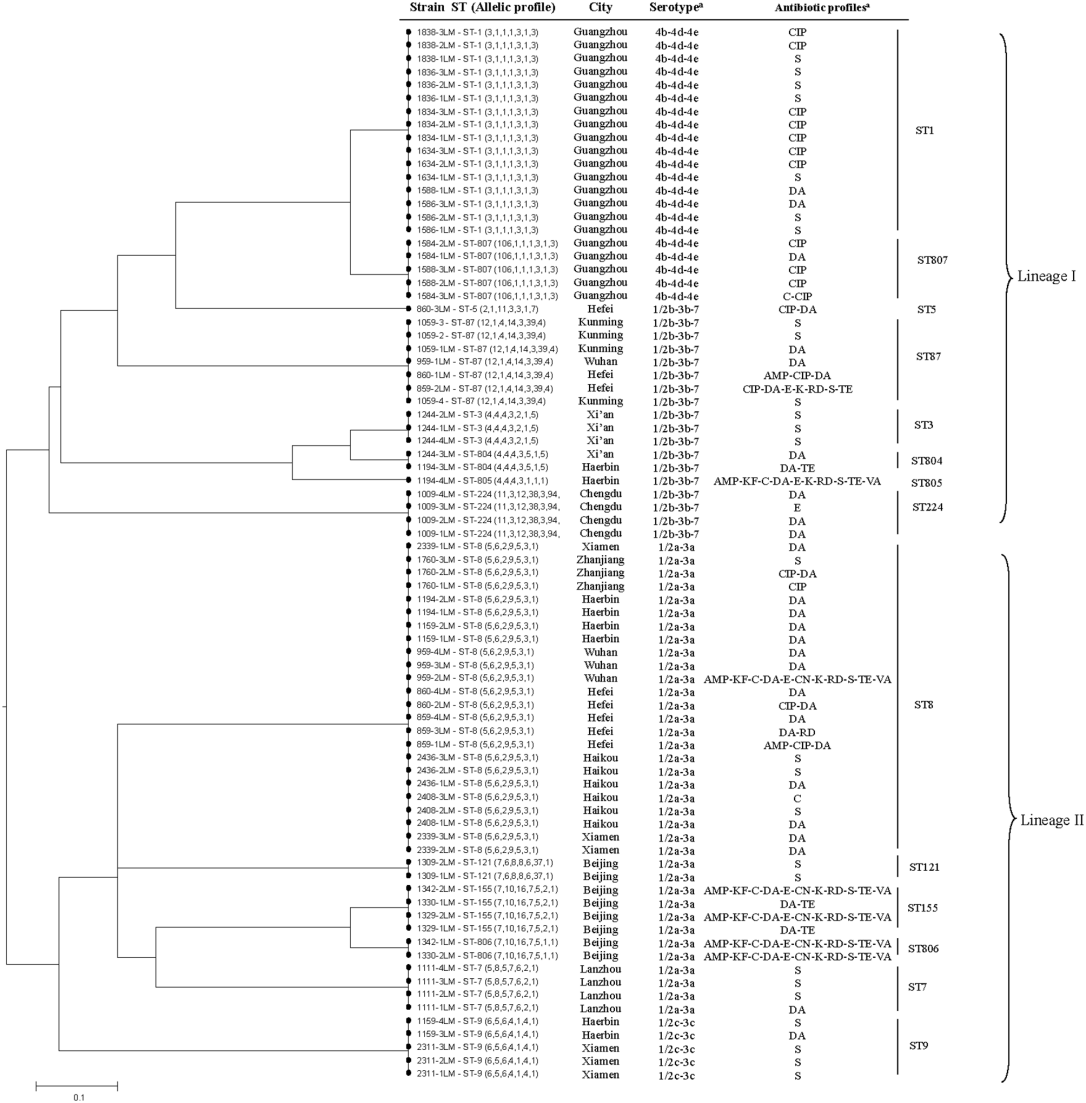
**The UPGMA (unweighted pair group method with arithmetic mean) tree of the seven multi-locus sequence typing loci (3288 base pair concatenated length) of RTE *L. monocytogenes* isolates (Supplementary Table 1)**. This tree was generated using the S.T.A.R.T (version 2).

MLST detected a total of 14 different sequence types in the 80 isolates, including four new STs (ST804, ST805, ST806, and ST807) (Table 2). The most common allelic profile was ST8 (24/80, 30% of isolates) followed by ST1 (16/80, 20% of isolates) independently of the isolates source. With the exception of ST5 and ST805, the remainder of the STs included more than one isolate. Of the STs, ST-87, ST5, ST224, ST804, ST805, and ST3 belonged to serovar II.2 (1/2b-3b-7), ST8, ST7, ST122, ST155, and ST806 belonged to serovar I.1 (1/2a-3a), ST1 and ST807 belonged to serovars II.1 (4b-4d-4e), and ST9 belonged to serovar I.2 (1/2c-3c). Based on each ST matches at least one other ST at ≥6 of the 7 loci, three clonal complexes (CCs) were identified, including CC1 (ST1, ST807), CC3 (ST3, ST804), and CC155 (ST155, ST806). A phylogenetic tree based on the seven concatenated MLST sequences (Figure 1) shows the relatedness between the RTE strains. STs correlated well with serotype and lineage. Most isolates recovered from same city were clustered into one type. Furthermore, it should be noted that the multidrug resistant strains 1194-4LM, 1330-2LM, and 1342-1LM were belonged to new STs (ST805, ST806) indicating that these isolates were genetically diverse from other isolates.

**Table 2:** **Allelic profile (STs) of RTE *L. monocytogenes* isolates for MLST**.

**STs**	**Profile**							**No. of isolates (%)**	**Serovar**
	***abcZ***	***bglA***	***cat***	***dapE***	***dat***	***Idh***	***lhkA***		
8	5	6	2	9	5	3	1	24 (30)	1/2a-3a
1	3	1	1	1	3	1	3	16 (20)	4b-4d-4e
87	12	1	4	14	3	39	4	7 (8.75)	1/2b-3b-7
9	6	5	6	4	1	4	1	5 (6.25)	1/2c-3c
807	106	1	1	1	3	1	3	5 (6.25)	4b-4d-4e
7	5	8	5	7	6	2	1	4 (5)	1/2a-3a
155	7	10	16	7	5	2	1	4 (5)	1/2a-3a
224	11	3	12	38	3	94	2	4 (5)	1/2b-3b-7
3	4	4	4	3	2	1	5	3 (3.75)	1/2b-3b-7
804	4	4	4	3	5	1	5	2 (2.5)	1/2b-3b-7
121	7	6	8	8	6	37	1	2 (2.5)	1/2a-3a
806	7	10	16	7	5	1	1	2 (2.5)	1/2a-3a
5	2	1	11	3	3	1	7	1 (1.25)	1/2b-3b-7
805	4	4	4	3	1	1	1	1 (1.25)	1/2b-3b-7

The evolutionary characteristics of the seven housekeeping genes among the isolates were analyzed using the software dnasp5, which can distinguish between randomly (i.e., neutrally) and non-randomly evolving DNA sequences. Tajima's D test indicated that *abcZ, cat, dapE, dat, ldh*, and *lhkA* evolved randomly, whereas *bglA* evolved non-significantly (0.9151, *P* > 0.10) (Table 3). However, seven gene portions harbored a total of 195 polymorphisms (5.93%; range 3.51–12.1% per gene). The average nucleotide diversity π was 2.6%, ranging from 0.9 to 6.2% per gene. The GC% observed in all alleles ranged from 36.4 to 42.9%, which was consistent with the 39% value observed across the entire *L. monocytogenes* EGDs genome (Glaser et al., 2001).

**Table 3:** **Polymorphism of seven housekeeping protein-coding genes among *L. monocytogenes* isolates**.

**Gene**	**Template size**	**No. (%) polymorphic sites**	**G+C content (%)**	**Ks**	**Ka**	**Ka/ks**	**π**	**Tajima's D**
*abcZ*	537	32 (5.96)	37.5	0.09824	0.00145	0.01476	0.02116	2.38645^*^
*bglA*	399	14 (3.51)	40.5	0.04069	0.00101	0.02482	0.00938	0.9151
*cat*	486	22 (4.53)	41.2	0.09135	0.00223	0.02441	0.0201	3.34927^***^
*dapE*	462	36 (7.79)	42.9	0.15169	0.0095	0.06263	0.03581	2.71776^***^
*dat*	471	57 (12.1)	36.4	0.31833	0.01483	0.04659	0.06114	4.53398^***^
*ldh*	453	17 (3.75)	43.4	0.06825	0	0.00000	0.01465	2.71776^**^
*lhkA*	480	17 (3.54)	37.1	0.07193	0.00273	0.03795	0.01693	3.98189^***^
Concatenate	3288	195 (5.93)	39.8	0.1116	0.00456	0.04086	0.02586	3.8258^***^

*Ks, number of synonymous mutations; Ka, number of non-synonymous mutations; π, Nucleotide diversity*.

* *Indicate statistical differences of P < 0.05*.

** *Indicate statistical differences of P < 0.01*.

*** *Statistical differences of P < 0.001*.

### Prevalence of virulence associated genes and EC markers

Eighty isolates of *L. monocytogenes* from retail RTE food were detected for the presence of virulence genes. The result showed that all isolates harbored listeriolysin O genes (*hly*) and internalin genes (*inlA, inlB, inlC*, and *inlJ*), and 38.8% (31/80) of isolates harbored listeriolysin S genes (*llsX*) (Table 4). The *llsX*-positive isolates including twenty-one 4b-4d-4e isolates, eleven 1/2b-3b-7 isolates. EC markers were identified in 26.3% (21/80) of isolates by multiplex PCR. ECI was the only EC marker identified and was observed in 4b-4d-4e isolates from roast meat (roast chicken/duck/pork) in this study. These EC isolates were also found to be positive for *llsX*.

**Table 4:** **Prevalence of virulence markers in 80 RTE *Listeria monocytogenes* strains**.

**Virulence markers**	**No. (%) of positive samples**	**No. (%) of serotype-positive isolates**				
		**1/2a-3a**	**1/2c-3c**	**4b-4d-4e**	**1/2b-3b-7**	**4a-4c**
*hly*	80/80 (100)	36 (100)	5 (100)	21 (100)	18 (100)	0 (0)
*inlB*	80/80 (100)	36 (100)	5 (100)	21 (100)	18 (100)	0 (0)
*inlA*	80/80 (100)	36 (100)	5 (100)	21 (100)	18 (100)	0 (0)
*inlC*	80/80 (100)	36 (100)	5 (100)	21 (100)	18 (100)	0 (0)
*inlJ*	80/80 (100)	36 (100)	5 (100)	21 (100)	18 (100)	0 (0)
*llsX*	31/80 (38.8)	0 (0)	0 (0)	21 (100)	10 (55.6)	0 (0)
ECI	21/80 (26.3)	0 (0)	0 (0)	21 (100)	0 (0)	0 (0)
ECII	0/80 (0)	0 (0)	0 (0)	0 (0)	0 (0)	0 (0)
ECIII	0/80 (0)	10 (8.9)	0 (0)	0 (0)	0 (0)	0 (0)

### Analysis of selected isolates for *inlA* sequence

Sequencing the full 2400 bp *inlA* ORF in 35 *L. monocytogenes* isolates yielded 14 different *inlA* allelic types, indicating 88.6% of gene diversity. A total of 126 (5.2%) sites were polymorphic, including 85 sites with synonymous substitutions and 41 sites with non-synonymous substitutions. The average number of nucleotide differences per site between two sequences (π) was 0.01907; the average number of nucleotide differences (*k*) was 45.834. The GC% observed in *inlA* was 37.1%.

**Figure 2:**
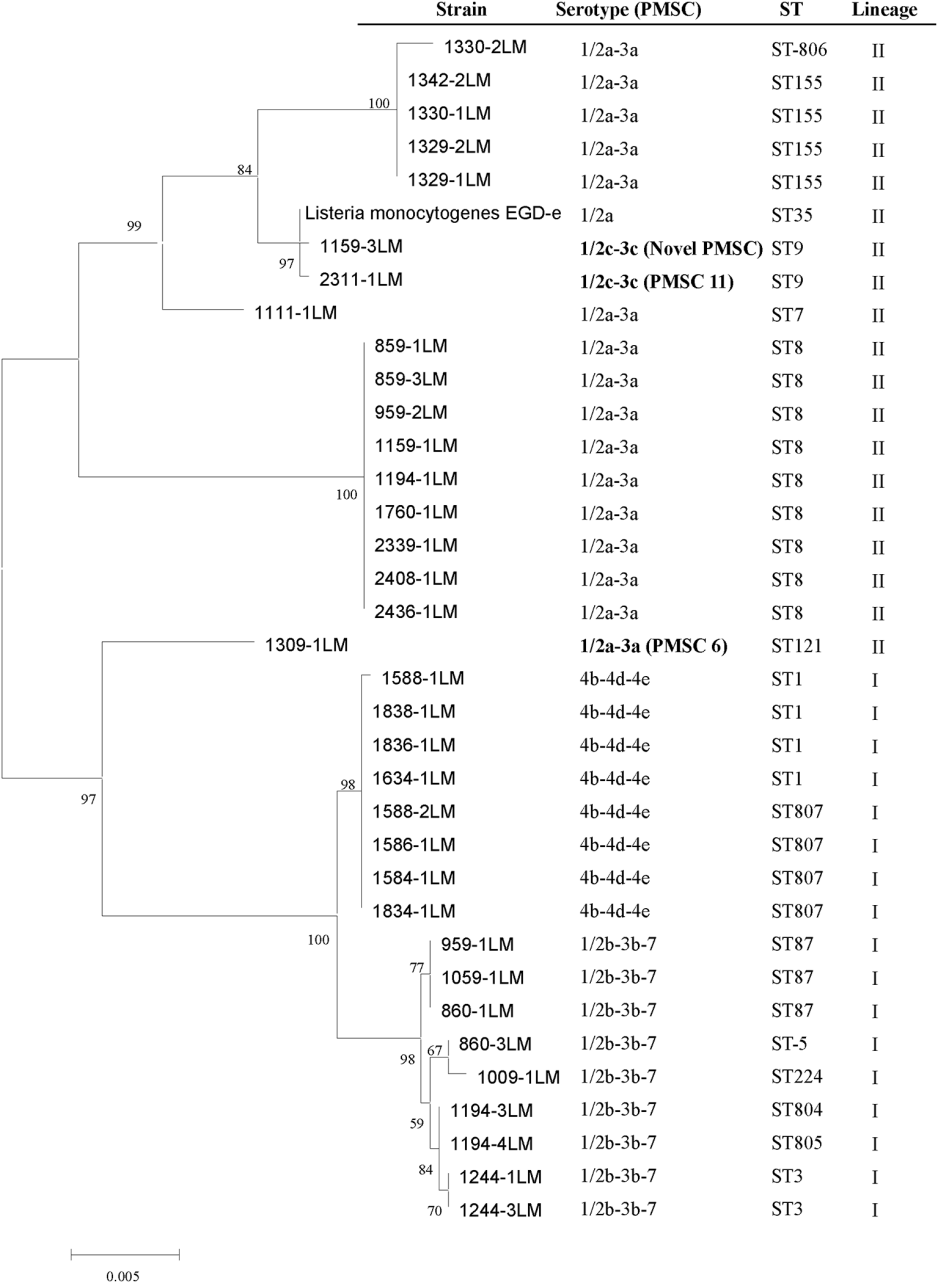
**Maximum likelihood tree of selected RTE *L. monocytogenes* isolates based on *inlA* sequence**. The *inlA* sequences of 36 isolates are shown, including 35 selected isolates in this study and *Listeria monocytogenes* EGDe.

The phylogenetic tree generated using the *inlA* sequences of 35 RTE isolates and the *L. monocytogenes* EGDe reference strain (NC_003210.1; serotype 1/2a, ST35) revealed a similar grouping based on MLST analysis (Figure 2). The same lineage exhibited close relationships. Truncated forms of *InlA* have been described and are associated with reduced virulence (Nightingale et al., 2005a; Van Stelten et al., 2010), three distinct *inlA* alleles were found to harbor PMSC at position 492 (PMSC type 6), carried by ST 121 (1309-1LM, 1/2a-3a), the mutation at position 685 (PMSC type 11) harbored by the serotype 1/2c-3c isolate (2311-1LM, ST9) and a nonsense mutation at position 326 (GAA → TAA) of the *inlA* gene where a change of glutamic acid codon to a stop codon occurs showed a novel mutation type of PMSC (1159-3LM, 1/2c-3c, ST9).

**Figure 3:**
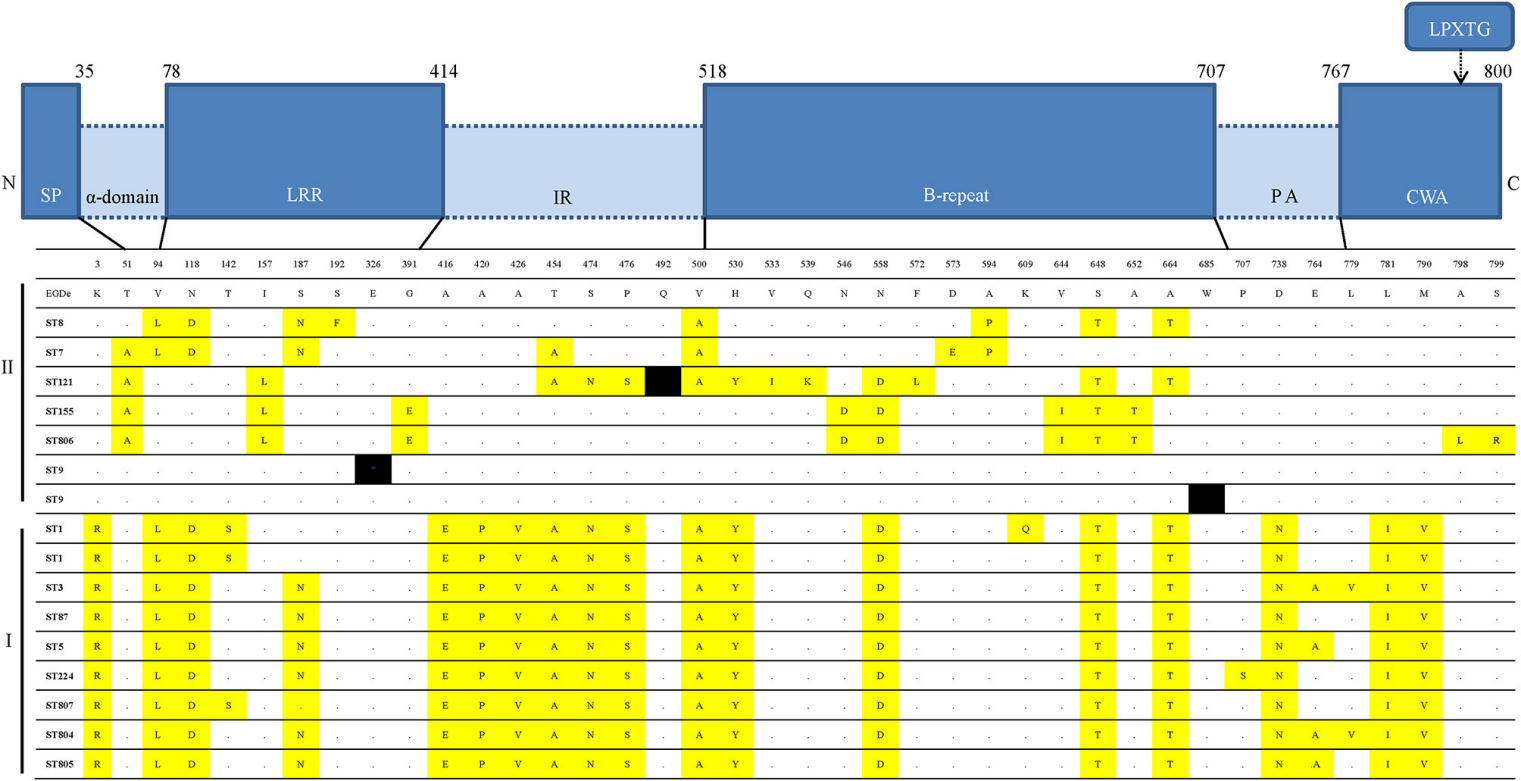
**Internalin A variants in the 16 distinct ST and *inlA* alleles encountered**. Comparison of the amino acid sequences of inlA of 35 *L. monocytogenes* isolated from Chinese RTE food to the homologous sequence of type strain EGDs. InlA functional domains are represented as distinct blocks: SP, signal peptide, α-domain, alpha-domain linker; LRRs, leucine rich repeats; IR, intragenic repeat, B-repeats, PA, pre-anchor domain; CWA, cell wall anchor; and C, C terminus. Yellow represents replacement with amino-acid and black indicates nonsense mutations leading to a premature stop codon (PMSC).

Comparison of the amino-acid sequences of *inlA* between 35 *L. monocytogenes* isolates and the homologous sequence type strain EGDs, 13 internalin A variants were grouped. Each STs showed same variants, excluding CC1 (ST1, ST807), CC3 (ST3, ST804), and ST5 and ST805. Isolates from ST1 divided into two variants. In total, 40 amino acids were substituted (40/800, 5%). Most of the substitutions (31/40, 77.5%) occurred in the leucine-rich repeats (LLR), the inter-repeat (IR) domain and the B-repeat domain (Figure 3). The most conserved region of InlA among the 35 RTE *L. monocytogenes* isolates was the LRR domain. In parallel, a previous study (Ragon et al., 2008) reported the high constraint of the LRR-region and the moderate constraint of the IR- and B-repeat regions.

## Discussion

As an important foodborne pathogen, *L. monocytogenes* remains a signification public health and food safety threat worldwide. In China, many studies of the prevalence of *L. monocytogenes* in food have been reported (Chen et al., 2009, 2014), but the outbreaks and infection of listeriosis in food are very limited. However, not all *L. monocytogenes* isolates have an equal capacity to cause disease. Therefore, it is important to investigate the molecular characteristics and virulence potential of *L. monocytogenes* isolates for designing and implementing more effective prevention strategies. The RTE isolates analyzed in this study were isolated from most of provincial capitals of China (Wu et al., 2015a), which could be better understanding the characterization of this important pathogen in China.

To enable increased characterization, MLST can be complemented by the nucleotide sequence determination of one or more highly diverse genes, such as those encoding antigens or antibiotic resistance determinants (Sullivan et al., 2005). In this study, 80 *L. monocytogenes* RTE isolates belonged to limited number of major clones. Except four new STs (ST804, 805, 806, 807), the other detected STs were already described in the *L. monocytogenes* Institute Pasteur MLST database (http://bigsdb.web.pasteur.fr/perl/bigsdb/bigsdb.pl?db=pubmlst_listeria_isolates_public&page=profiles): 327 strains of ST1 (51% from human isolates), 253 strains of ST3 (37% from human and 55% from food and environment), 143 strains of ST7 (63% from animal and feed), 37 strains of ST8 (including 15 human and 10 food), 130 strains of ST9 (35% from human and 21% from food), 52 strains of ST5 (50% from human and 23% from food), 22 strains of ST87 (55% from human and 23% from food), 70 strains of ST155 (50% from human), 6 strains of ST224 (2 isolates from human and 4 from environment), and 74 strains of ST121 (54% from food). Strains of all STs observed in our study have been associated to various extents with human listeriosis and outbreaks, indicating that *L. monocytogenes* strains of these STs have at least a theoretical pathogenic potential. It is worth nothing that ST1, comprising 20% of the isolates in this study, have the same sequences as F2365 (a serotype 4b isolates from a soft cheese outbreak in California in 1985), CLIP12848 (a serotype 4b isolates from outbreak in France in 1989), and CLIP68868 (a serotype 4b isolates from outbreak in Sweden 1995) which should the worthy of the attention of the food hygiene supervision department. Interestingly, novel STs (ST805, ST806) were found in multidrug resistant strains (Figure 1), showing some allele numbers had exchanged. Multiple resistance in *L. monocytogenes* was linked to the presence of a self-transferable plasmid that was proposed to originate in *Enterococcus*-*Streptococcus* (Charpentier and Courvalin, 1999). Therefore, the self-transferable plasmid may also impact the homologous recombination of housekeeping genes. However, further research is needed to determine the reason underlying this correlation.

The presence of *hly, inlA, inlB, inlC, inlJ*, and *llsX* was evaluated in *L. monocytogenes* isolates recovered from patients, food and the environment (Kathariou, 2003; Wieczorek et al., 2012; Wu et al., 2015b). In this study, PCR based analysis of the 80 RTE isolates showed that most of these isolates possessed virulence genes similar to those of clinical isolates (Mammina et al., 2009). The *hly* gene is encoding the listeriolysin O (LLO), the determinant that is required for the disruption of the phagocytic vacuole and the release of bacteria into the cytoplasm, a prerequisite for their intracellular proliferation. Therefore, LLO is an essential virulence factor and its absence leads to total avirulence (Portnoy et al., 1992). *L. monocytogenes* adheres to and actively through actions of internalins, a complex family of LRR-containing proteins (Bierne et al., 2007). In this study, we selected *inlA, inlB, inlC*, and *inlJ* for tested internalins in *L. monocytogenes* isolates. These genes are claimed to play a role in pathogenesis of human listeriosis. Besides, the *llsX* gene (encoding LLS) was employed as a genetic marker to detect *L. monocytogenes* with *Listeria* pathogenicity island 3 (LIPI-3) (Cotter et al., 2008), and strains from the present study exhibited 38.8% (31/80) positivity. The LLS, which is present in a subset of strains of lineage I, is induced only under oxidative stress conditions and contributes to murine virulence and in survival in polymorphonuclear neutrophils (Cotter et al., 2008). Overall, our result showed that the positive-*llsX* strains were found exclusively in lineage I (shown Table 3 and Figure 1), the evolutionary lineage of *L. monocytogenes* that contributes to the majority of spontaneous and epidemic outbreaks of listeriosis (Jeffers et al., 2001). Besides, most of positive-*llsX* strains involving ECI (21/32, 65.6%) have been related to several major outbreaks in the US and Europe in the past (Kathariou, 2003; Liu, 2008). In general, ECs is defined as a small number of isolates of a presumably common ancestor (Chen and Knabel, 2007). Specific ECs continue to be associated with sporadic cases around the world (Neves et al., 2008; Mammina et al., 2009; Lomonaco et al., 2013). Recently, Cantinelli et al. (2013) conclude that the “epidemic clone” denominations represent a redundant but largely incomplete nomenclature system for MLST-defined clones, which must be regarded as successful genetic groups that are widely distributed across time and space. In our study, *L. monocytogenes* isolates of MLST-CC1, including ST1 and ST807 were both ECI which was consistent with this conclusion. However, the high occurrence of multiple virulence-associated genes and ECI markers in *L. monocytogenes* isolates from Chinese RTE food could pose a significant health risk as these isolates can be pathogenic and potentially capable of causing an epidemic.

Certainly, the presence of these virulence-associated genes does not mean a particular strain is virulent, as these genes are normally present in *L. monocytogenes* but if absence of these genes lead to avirulence. Over the past decade, previous studies identified naturally occurring PMSC mutations in *inlA* and have demonstrated that these mutations are responsible for virulence attenuation (Van Stelten and Nightingale, 2008). *L. monocytogenes* strains with PMSCs were frequently isolated from RTE foods in the US, although *inlA* PMSCs were markedly underrepresented among ECs (Van Stelten et al., 2010). In our study, the sequencing of *inlA* genes from isolates revealed that three mutations resulted in PMSCs: type 6, 11 and a novel type at position 326 (GAA → TAA), which means most of strains (32/35, 91.4%) are capable of producing full-length InlA required for host cell invasion. Generally, PMSCs have been reported 30%-45% of prevalence in food but 5% of prevalence in clinical isolates (Jacquet et al., 2004; Van Stelten et al., 2010; Chen et al., 2011). Three mutations were both harbored by the lineage II strains (2 strains with ST9, one strain with ST121) (Figure 2), which further confirmed that 1/2a and 1/2c (lineage II) more frequently possess PMSCs than 1/2b or 3b serotypes, with 4b strains rarely having PMSCs (Orsi et al., 2011). PMSC seems to be more frequently observed in ST9 and ST121 isolates as previous reports (Ragon et al., 2008; Ciolacu et al., 2014). ST9 and ST121 include isolates from several different countries and from several different environmental, clinical and food source (Ragon et al., 2008; Parisi et al., 2010; Holch et al., 2013). Some *L. monocytogenes* strains can persist for a longtime in food processing. The repeatedly lost by convergent evolution in the genetically homogeneous of these genotypes, may be attribute to the selective advantage by the loss of a functional InlA protein or a relaxed selective constraint on maintaining InlA function (Ragon et al., 2008). It is worth mentioning that the novel PMSCs type at position 326 (GAA → TAA), indicated that variation occurred in the LLR domain were located in repeats 10 (Figure 3). This domain promotes interaction with human surface receptor, E-cadherin, and has been reported as highly conserved, especially from repeats 7 to 15 (Ragon et al., 2008). To date, at least 18 distinct mutations in *inlA* leading to PMSCs have been observed in *L. monocytogenes* isolates worldwide, only four of them (type 5, 16, 17, and 18) were found in LLR (Van Stelten et al., 2010). Compared with the inlA variants, *L. monocytogenes* retained the ability for localized recombination is clearly provided by the *inlA* gene coding InlA, in agreement with previous reports (Nightingale et al., 2005b; Orsi et al., 2007). The substitution of some amino acid were consistent between lineage I strains and II, such as lysine replaced into arginine at position 3 in all lineage I strains (Figure 3). Additionally, the close relationship between *inlA* sequencing and MLST typing (Figure 2) suggested that housekeeping genes and virulence-associated genes had some similar genetic characterization, representing monophyletic origin. In other words, our data suggest that *L. monocytogenes* evolved and generated different STs related to some degree of evolutionary consistent.

In summary, the research findings suggest that *L. monocytogenes* isolated from Chinese RTE food showed genetic relatedness and virulence attribute. Serotype, lineage, MLST types, even antibiotic resistance showed some degree of genetically relationship. Most MLST sequence types (10/14, 71.4%) found in this study have been linked to human listeriosis around the world. All isolates carried several important virulence genes (*inlA, inlB, inlC, inlJ*, and *hly*), whereas 38.8% (31/80) of strains harbored listeriolysin S genes (*llsX*). Besides, all 4b-4d-4e isolates belonged to ECI showed a high potential to cause human diseases. Further study of *inlA* sequencing showed most of selected strains (32/35, 91.4%) contained PMSC-lacking *inlA* gene sequences required for the encoding of InlA factor and bacterial invasion of the host cell. Based on the genetic properties observed in *L. monocytogenes* isolates, it is reasonable to assume *L. monocytogenes* found in Chinese retail RTE food have potential to cause listeriosis, and is concerning. These data have significant implications for the epidemiological and public health studies of this pathogen.

## Author contributions

Conceived and designed the experiments: QW, JZ, SW. Performed the experiments: SW. Analyzed the data: SW, MC. Contributed reagents/materials/analysis tools: WG. Contributed to the writing of the manuscript: SW, QW.

## Funding

We would like to acknowledge the financing support of National Natural Science Foundation of China (No. 31471664), the Science and Technology Projects of Guangdong (2013B050800026), and the Science and Technology Projects of Guangzhou (201504010036).

### Conflict of interest statement

The authors declare that the research was conducted in the absence of any commercial or financial relationships that could be construed as a potential conflict of interest. The reviewer RT and handling Editor declared a current collaboration and the handling Editor states that the process nevertheless met the standards of a fair and objective review.
