# Supplementary material for: Analysis of Multilocus Sequence Typing and Virulence Characterization of Listeria monocytogenes Isolates from Chinese Retail Ready-to-Eat Food
Source: Front Microbiol. 2016 Feb 16;7:168. doi: 10.3389/fmicb.2016.00168 (PMC4754575; doi:10.3389/fmicb.2016.00168)
Supplement: Supplementary file 1 [file Table1.DOCX]

Supplementary Material

Analysis of Multilocus Sequence Typing and Virulence Characterization of *Listeria monocytogenes* Isolates from Chinese Retail Ready-to-eat Food

**Shi Wu, Qingping Wu^*^, Jumei Zhang, Moutong Chen, Weipeng Guo**

*** Dr. Qingping Wu:** [**wuqp203@163.com**](mailto:wuqp203@163.com)

**Supplementary Table 1.** Primer sequences, PCR preparations and conditions used for *L. monocytogenes* isolates in this study

| Gene target | Primer sequences (5’-3’) | Product sizes(bp) | PCR preparation | PCR condition | References |
| --- | --- | --- | --- | --- | --- |
| *inlB* | F:GATATTGTGCCACTTTCAGGTT  R: CCTCTTTCAGTGGTTGGGTT | 367 | 10 μl 2× DreamTaq mastermix, 7 μl nuclease free water, 80 ng template DNA, and 0.06 μM of each primer | 5 min at 94°C, 35 cycles of 94 °C for 35 s, 60 °C for 45 s, 72 °C for 1 min and a final extension at 72 °C for 10 min | Xu et al. (2009) |
| *hly* | F:GTTAATGAACCTACAAGACCTTCC  R: ACCGTTCTCCACCATTCCCA | 707 |  |  |  |
| *inlA* | F: ACGAGTAACGGGACAAATGC  R: CCCGACAGTGGTGCTAGATT | 800 | 12.5 μl 2× DreamTaq mastermix, 6 μl nuclease free water, 80 ng template DNA, and 0.4 μM *inlA* primer, 0.3 μM *inlC* primer, and 0.2 μM *inlJ* primer | 2 min at 94°C, 30 cycles of 94 °C for 30 s, 55 °C for 30 s, 72 °C for 1 min and a final extension at 72 °C for 10 min | Liu et al. (2007) |
| *inlC* | F: AATTCCCACAGGACACAACC  R: CGGGAATGCAATTTTTCACTA | 517 |  |  |  |
| *inlJ* | F: TGTAACCCCGCTTACACACAGTT  R: AGCGGCTTGGCAGTCTAATA | 238 |  |  |  |
| *llsX* | F: TTATTGCATCAATTGTTCTAGGG  R: CCCCTATAAACATCATGCTAGTG | 200 | 12.5 μl 2× DreamTaq mastermix, 3.5 μl nuclease free water, 80 ng template DNA, and 0.4 μM primer | 3 min at 95°C, 45 cycles of 95 °C for 30 s, 60 °C for 1 min, 72 °C for 1 min and a final extension at 72 °C for 10 min | Clayton et al., 2011 |
| ECI | F: AATAGAAATAAGCGGAAGTGT  R: TTATTTCCTGTCGGCTTAG | 303 | 12.5 μl 2× DreamTaq mastermix, 6 μl nuclease free water, 80 ng template DNA, and 0.4 μM ECI primer, 0.7 μM ECII primer, and 0.3 μM ECIII primer | 3 min at 95°C prior to 15 cycles of 1 min at 94°C, 1 min with a touchdown from 55°C to 51°C (3 cycles per temperature), and 1 min at 72°C, followed by 15 cycles of 1 min at 94°C, 1 min at 50°C, and 1 min at 72°C and 1 final cycle for 8 min at 72°C | Chen & Knabel (2007) |
| ECII | F: ATTATGCCAAGTGGTTACGGA  R: ATCTGTTTGCGAGACCGTGTC | 889 |  |  |  |
| ECIII | F: TTGCTAATTCTGATGCGTTGG  R: GCGCTAGGGAATAGTAAAGG | 497 |  |  |  |
