# Supplementary material for: Analysis of Multilocus Sequence Typing and Virulence Characterization of Listeria monocytogenes Isolates from Chinese Retail Ready-to-Eat Food
Source: Front Microbiol. 2016 Feb 16;7:168. doi: 10.3389/fmicb.2016.00168 (PMC4754575; doi:10.3389/fmicb.2016.00168)
Supplement: Supplementary file 2 [file Table2.DOCX]

Supplementary Material

Analysis of Multilocus Sequence Typing and Virulence Characterization of *Listeria monocytogenes* Isolates from Chinese Retail Ready-to-eat Food

**Shi Wu, Qingping Wu^*^, Jumei Zhang, Moutong Chen, Weipeng Guo**

*** Dr. Qingping Wu:** [**wuqp203@163.com**](mailto:wuqp203@163.com)

**Supplementary Table 2.** Primers used for *inlA* PCR and sequencing

|  | Primer | Primer sequences (5’-3’) | Annealing temperature (°C) |
| --- | --- | --- | --- |
| Amplification primers | inlA F | CGGATGCAGGAGAAAATCC | 55 |
|  | inlA R | CTTTCACACTATCCTCTCC |  |
| Sequencing primers | CK_0814292_inlA.R | TAGCCAGAACACTAATATCC | - |
|  | CK_0814293_inlA.F1 | TGACGAATCTAACTGGTTTG | - |
|  | CK_0814294_inlA.F2 | AACAAGGTAAGTGACGTAAG | - |
|  | CK_0814295_inlA.F3 | CAAGAACCTACGGCACCAAC | - |
|  | HC_0817387_inlA.F3new^a^ | GTGGTGACAAGTGGGATTTC | - |

a 1111-1LM, 1159-3LM, 1329-1LM, 1329-2LM, 1330-1LM, 1330-2LM, 1342-2LM were used this primer for sequencing.
